# 5-Nitro-3-(2-(4-phenylthiazol-2-yl)hydrazineylidene)indolin-2-one derivatives inhibit HIV-1 replication by a multitarget mechanism of action

**DOI:** 10.3389/fcimb.2023.1193280

**Published:** 2023-06-23

**Authors:** Angela Corona, Rita Meleddu, Olivier Delelis, Frederic Subra, Filippo Cottiglia, Francesca Esposito, Simona Distinto, Elias Maccioni, Enzo Tramontano

**Affiliations:** ^1^ Department of Life and Environmental Sciences, University of Cagliari, Monserrato, Italy; ^2^ Laboratory of Biology and Applied Pharmacology (LBPA), Ecole Normale Supérieure (ENS) Cachan, Centre National de la Recherche Scientifique (CNRS), Cachan, France

**Keywords:** antiviral agents, drug design, multitarget inhibitors, RT inhibitors, IN inhibitors

## Abstract

In the effort to identify and develop new HIV-1 inhibitors endowed with innovative mechanisms, we focused our attention on the possibility to target more than one viral encoded enzymatic function with a single molecule. In this respect, we have previously identified by virtual screening a new indolinone-based scaffold for dual allosteric inhibitors targeting both reverse transcriptase-associated functions: polymerase and RNase H. Pursuing with the structural optimization of these dual inhibitors, we synthesized a series of 35 new 3-[2-(4-aryl-1,3-thiazol-2-ylidene)hydrazin-1-ylidene]1-indol-2-one and 3-[3-methyl-4-arylthiazol-2-ylidene)hydrazine-1-ylidene)indolin-2-one derivatives, which maintain their dual inhibitory activity in the low micromolar range. Interestingly, compounds 1a, 3a, 10a, and 9b are able to block HIV-1 replication with EC_50_ < 20 µM. Mechanism of action studies showed that such compounds could block HIV-1 integrase. In particular, compound 10a is the most promising for further multitarget compound development.

## Introduction

Since the identification of the human immunodeficiency virus (HIV) as the etiologic agent of acquired immunodeficiency syndrome (AIDS) (Barré-Sinoussi et al., 1983), the efforts of the scientific community have led to the approval of an originally unpredictable number of drugs belonging to six different classes, which, combined in several therapeutic regimens called highly active antiretroviral therapy (HAART), represent a unique success in the treatment of viral infections, turning a highly lethal syndrome into a chronic disease. HIV-1 infects CD4+ cells of the immune system, causing persistent infection. Moreover, it integrates its genome into the host cell genome and replicates, thus requiring a permanent treatment to control the viremia and to avoid progression to AIDS.

The management of this disease, however, is very difficult, and AIDS-related numbers are still impressively high, with 38.4 million people living with HIV, 1.5 million newly infected, and 650,000 deaths in 2021. Although AIDS-related mortality was reduced by the introduction of HAART, all the drugs on the market are susceptible to the selection of drug-resistant variants (Gupta et al., 2018), even the new first-line integrase (IN) inhibitors. The scientific community’s efforts are focused on the identification of innovative compounds able to block the circulating drug-resistant variants. In this context, the identification of agents capable of simultaneously blocking multiple viral functions (multitarget approach) represents a very promising strategy for the treatment of HIV (Ramsay et al., 2018). As a result, the replicative cycle of HIV has been studied in detail, and many of its steps have been validated as pharmacological targets (Sierra and Walter, 2012; Puhl et al., 2019).

In particular, two proteins play a key role in the early phases of the replication, namely, the reverse transcriptase (RT) (which converts the viral genome (ssRNA) into a proviral double-stranded DNA (dsDNA) (Sarafianos et al., 2009)) and the IN (which imports the viral genome into the host cell nucleus and integrates it into the host genome (Esposito and Craigie, 1999)). Once integrated, the provirus becomes a source of mRNAs that code for viral proteins and ssRNA genomes that together form the new viral particles.

RT is a multifunctional complex composed of two subunits of 66 and 51 kDa (p66/p51), with activities of DNA polymerase and ribonuclease H (RNase H). For DNA polymerization, RT uses either RNA (RNA-dependent DNA polymerase (RDDP) activity) or DNA (DNA-dependent DNA polymerase (DDDP) activity) as a template. The reverse transcription proceeds through an RNA/DNA hybrid, whose RNA must be removed to allow the synthesis of the second DNA strand. This process is operated by the RNase H-associated function through a sequence of highly specific hydrolytic events. Nevertheless, RT is extremely error-prone due to a missing draft control system, and the produced mutations can be amplified by recombination. This is one of the main reasons why the discovery of a vaccine is a hard task. Accordingly, due to their key role in viral replication, RDDP and RNase H are both valid targets for the identification of new inhibitors since both are essential to accomplish the reverse transcription, and none of them can be complemented by host proteins (Cristofaro et al., 2002; Corona et al., 2014a; Corona et al., 2014b).

However, all currently approved RT inhibitors only target the polymerase function, acting on the incorporation of nucleotides either as nucleoside analogs [nucleoside reverse transcriptase inhibitors (NRTIs)] or as non-nucleoside allosteric drugs [non-nucleoside reverse transcriptase inhibitors (NNRTIs)]. So far, no inhibitors of the RNase H function have been approved for clinical use. Thus, RNase H is the only viral enzyme function for which no inhibitor has undergone clinical development so far, making it a very promising and coveted target for the development of antiretroviral agents.

The DNA polymerase and RNase H activities are performed by two different domains on the p66 subunit. The DNA polymerase domain is located at the N-terminal and possesses the conformation of a “right hand”; conversely, the RNase H domain is located at the C-terminal portion, 60 Å far from the polymerase site. These two domains are connected by a connection domain. Long-range effects and a functional interdependence between the two active domains have been suggested, based on direct site mutagenesis studies that demonstrated how changes in the HIV-1 RT polymerase domain can influence the activity of RNase H, while deletions at the C terminal can decrease the efficiency of DNA polymerization (Li et al., 2016). This structural and functional interdependence is also supported by evidence showing that mutations in the RNase H domain influence resistance to NRTIs, while NNRTIs, such as nevirapine and efavirenz (EFV), increase the activity of the RNase H function (Julias et al., 2002; Corona et al., 2016b). In this scenario of close interdependence, the identification of a compound with the ability to inhibit both RT activities could represent a significant advance in the fight against drug resistance (Distinto et al., 2012; Distinto et al., 2013).

Over the years, three classes of RNase H inhibitors have been identified in preclinical studies: i) active site inhibitors, which chelate the two Mg^2+^ ions coordinated in the enzyme site by the DDE motif and used as cofactors in the catalysis; ii) allosteric inhibitors that bind the p66/p51 interface; and iii) dual allosteric inhibitors of both the RDDP and RNase H functions. None of them has reached clinical trials so far (Distinto et al., 2013; Corona et al., 2016a; Boyer et al., 2018; Martín-Alonso et al., 2022; Zhang et al., 2022).

Several HIV-1 RT RNase H inhibitors mostly belonging to active-site inhibitors showed promising activity against viral replication, with EC_50_ values in the low micromolar range (Corona et al., 2020; Kang et al., 2023). Although some reports suggested that RNase H inhibitors could negatively impact the activity of NRTIs such as AZT (Davis et al., 2011), it is worthy of note that some RNase H inhibitors were also reported to retain full potency of inhibition against HIV-1 vectors that carry well-known INSTI, NNRTI, and NRTI resistance mutations in a single round of replication (Boyer et al., 2018) and have full potency against HIV-1 K103N-Y181C replicant virus conferring cytoprotective effect in a 5 days of cytopathic effect (CPE) assay (Corona et al., 2020), making them very appealing as potential therapeutics to treat the circulating drug-resistant strains. Allosteric inhibition of RNase H/RDDP has been described for scaffolds characterized by variously substituted aromatic portions linked by a functionalized spacer containing groups capable of donating or accepting hydrogen bonds, whether linear or made from heterocyclic rings (Distinto et al., 2013; Meleddu et al., 2014; Meleddu et al., 2015; Meleddu et al., 2017; Sonar et al., 2017; Esposito et al., 2019; Fois et al., 2021; Meleddu et al., 2021). It has been proposed that these compounds possess an innovative mechanism of action since they could bind two different allosteric RT pockets: the first one is located at the DNA polymerase domain (partially overlapping the NNRTI binding pocket), and the second one is under the active RNase site H near the interface between p66/p51 (Corona et al., 2016a).

Interestingly, both the catalytic sites of RNase H and IN belong to the polynucleotidyl transferase superfamily and exhibit a high degree of structural similarity, suggesting the development of dual-action drugs targeting both proteins (Yang and Steitz, 1995; Esposito et al., 2020).

HIV-1 IN is composed of three structurally distinct domains: the N-terminal domain (NTD), the catalytic domain (CCD) (which contains the catalytic DDE motif and coordinates two Mg^2+^ ions), and the C-terminal domain (CTD) (Esposito and Tramontano, 2014). IN acts in the context of a macromolecular assembly called the pre-integration complex, which is made up of viral DNA, viral proteins, and host cell proteins. It performs two different catalytic reactions. In the cytoplasm, IN processes the ends of the viral DNA 3′ (3′-processing), while in the nucleus, this viral DNA is a substrate for the transfer reaction (stand transfer) and is inserted into the chromosome. IN acts as a multimer: the dimeric form is required for the 3′ processing phase, while the tetrameric form forms, with the processed DNA, a stable complex called the intasome, allowing the strand transfer reaction, which takes place through the interaction with a cellular factor: the human lens epithelium-derived growth factor (LEDGF/p75) (Esposito and Craigie, 1999).

**Figure 1:**
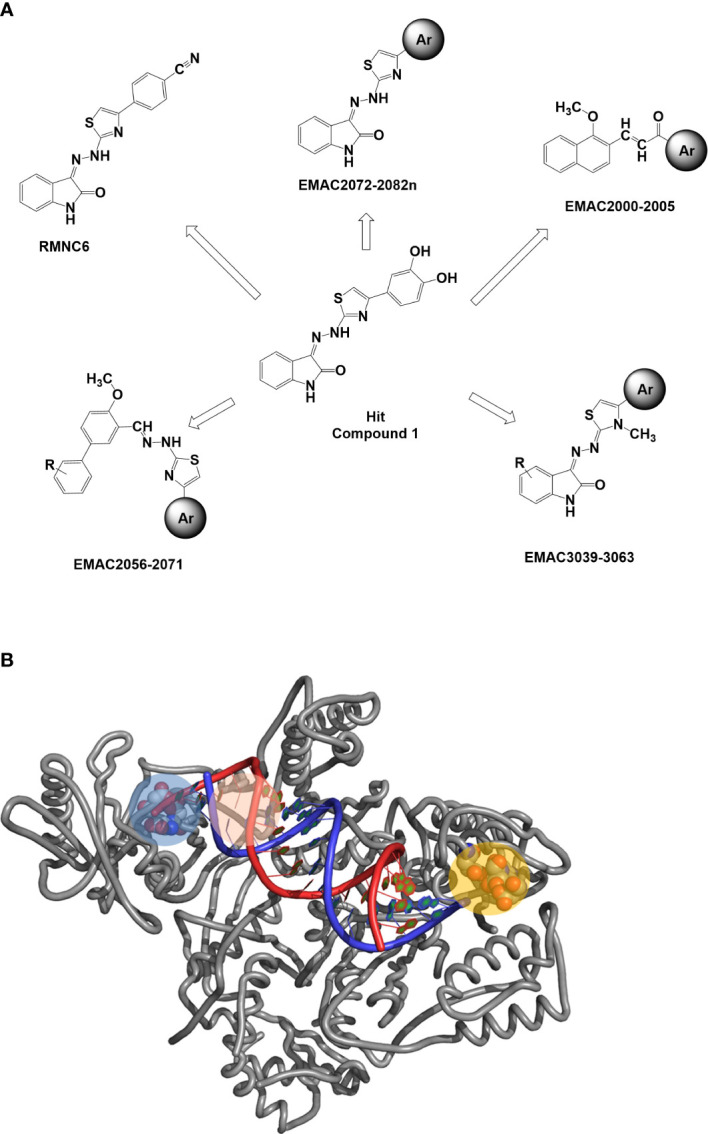
**(A)** Previously reported allosteric HIV RT dual inhibitors (Distinto et al., 2012; Meleddu et al., 2014; Meleddu et al., 2015; Corona et al., 2016a; Meleddu et al., 2017; Meleddu et al., 2021). Ar, aromatic substituents. **(B)** RT-HIV-1 and RDDP catalytic pocket in blue, RNase H and NNRTI allosteric pocket in pink, and RNase H catalytic pocket in orange. RT, reverse transcriptase; RDDP, RNA-dependent DNA polymerase; NNRTI, non-nucleoside reverse transcriptase inhibitor.

Inhibitors of IN represent the latest innovation in the repertoire of drugs for the treatment of HIV-1. Those approved so far, raltegravir (RAL), elvitegravir (EVG), and dolutegravir (DGV), all act in the active site of IN simultaneously interacting with the protein, the Mg cofactors, and the DNA by inhibiting the strand transfer reaction (INSTI). Unfortunately, although extremely effective in the management of HIV, all INSTIs are susceptible to failure through the selection of mutations that often also confer cross-resistance (Malet et al., 2015; Dekker José et al., 2022; Richetta et al., 2022; Dekker et al., 2023). For this reason, the interest of the scientific community is growing in the identification of allosteric inhibitors. Recently, molecular inhibitors of the IN site that binds LEDGF/p75 (LEDGIN), which block HIV-1 replication and modulate the oligomerization of IN, have been identified (Demeulemeester et al., 2014). Furthermore, a different single compound, Kuwanon-L, has been reported to bind a second allosteric site, inhibiting the catalytic activity of the enzyme and the interaction IN/LEDGF/p75, promoting and stabilizing the multimerization of IN, reducing its flexibility, and ultimately leading to the inhibition of the catalytic process (Esposito et al., 2015). Recently, it has been shown that Kuwanon-L also inhibits the activity of RT (Martini et al., 2017), opening the way to the development of dual allosteric inhibitors that inhibit both RT and IN. Thanks to the availability in public databases of structural information arising from both RT 3D high-resolution crystalline structures and ligands, both the ligand-based approach and the structure-based approach have been widely used to identify *in silico* new ligands (Frey et al., 2022). In addition, the resolution of the HIV-1 intasome (Passos et al., 2017; Engelman and Cherepanov, 2021) and the availability of several structures containing INSTI complexed to the Prototype Foamyvirus intasome, which presents a very high level of structural homology with HIV-1, make the study of molecules acting on both systems possible. The ((*Z*)-3-(2-(4-(3,4-dihydroxyphenyl)thiazol-2-yl)hydrazineylidene)indolin-2-one (1, **Figure 1**) structure was identified as the initial prototype for dual RT inhibitors through an *in silico* screening based on the shape-based method (Distinto et al., 2012). On these bases, the design and synthesis of differently substituted analogs were performed, which demonstrated a stable inhibitory capacity toward both RNase H and RDDP (Meleddu et al., 2014; Meleddu et al., 2015; Corona et al., 2016b; Meleddu et al., 2017; Meleddu et al., 2021) (**Figure 1**).

## Materials and methods

### Chemistry

Starting materials and reagents were obtained from commercial suppliers and were used without purification. All melting points were determined on a Stuart SMP11 melting points apparatus (Stone, UK) and were uncorrected. ^1^H-NMR was registered on a Bruker 500 MHz. All samples were measured in dimethyl sulfoxide (DMSO). Chemical shifts were reported in reference to the solvent in which they were measured. Coupling constants J were expressed in hertz (Hz). Elemental analyses were obtained on a Perkin-Elmer 240 B microanalyzer (Waltham, MA, USA). The analytical data of the synthesized compounds agreed within ±0.4% of the theoretical values. Thin-layer chromatography (TLC) was performed using silica gel plates (Merck F-254, Billerica, MA, USA), and spots were visualized by UV light.

### General procedure for the synthesis of compounds 1a–12a

A solution of equimolar amounts of 5-nitroisatin and hydrazinecarbothioamide in 2-propanol was refluxed in the presence of catalytic amounts of acetic acid. The reaction was monitored by TLC (ethyl-acetate/hexane = 1/1) until completion. The mixture was cooled to room temperature, and the solvent was removed under vacuum. The crude product was purified by column chromatography to give the desired 2-(5-nitro-2-oxoindolin-3-ylidene)hydrazine-1-carbothioamide with a yield of 97%; Rf = 0.28 (ethyl-acetate/hexane = 1/1); MS (ESI+): 266.03 [M+H]^+^.

The purified carbothioamide was then reacted with the appropriate α-halogen aryl-ketone in refluxing 2-propanol. The reaction was monitored by TLC until completion and allowed to cool to rt. A precipitate was formed, which was purified by crystallization (water/ethanol). By this procedure, compounds 1a–12a were synthesized (**Table 1**).

**Table 1:** Analytical data of derivatives 1a–12a.

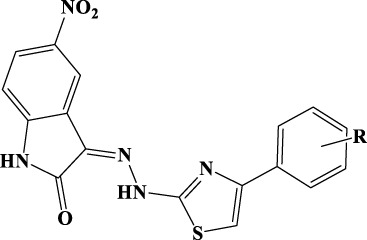					
Comp.	R	% Yield	CHN	^1^H-NMR	
			Calc./found		
**1a**	4-Cl	63	51.07; 2.52; 17.52 51.O8; 2.51; 17.51	^1^H-NMR (DMSO) δH 13.25 (brs, 1H, NH), 12.22 (s, 1H, NH), 8.6 (s, 1H, Ar-CH), 7.92 (d, 2H, J: 9, Ar-CH), 7.87 (d, 2H, J: 9, Ar-CH), 7.76 (s, 1H, thiazole), 7.48 (d, 1H, J: 8.5, Ar-CH), 7.11 (d, 1H, J: 8.5, Ar-CH)	
**2a**	4-F	56	53.26; 2.63; 18.27 53.27; 2.62; 18.26	^1^H-NMR (DMSO) δH 13. 23 (brs, 1H, NH), 12.22 (s, 1H, NH), 8.6 (s, 1H, Ar-CH), 8.25 (d, 2H, J: 9, Ar-CH), 7.69 (s, 1H, thiazole), 7.35 (d, 2H, J: 9, Ar-CH), 7.48 (d, 1H, J: 8.5, Ar-CH), 7.11 (d, 1H, J: 8.5, Ar-CH)	
**3a**	4-Br	61	45.96; 2.27; 15.76 45.95; 2.27; 15.75	^1^H-NMR (DMSO) δH 13.25 (brs, 1H, NH), 12.22 (s, 1H, NH), 8.6 (s, 1H, Ar-CH), 8.22 (d, 2H, J: 9, Ar-CH), 7.86 (d, 2H, J: 9, Ar-CH), 7.77 (s, 1H, thiazole), 7.50 (d, 1H, J: 8.5, Ar-CH), 7.12 (d, 1H, J: 8.5, Ar-CH)	
**4a**	4-NO_2_	67.5	49.76; 2.46; 20.48 49.75; 2.46; 20.47	^1^H-NMR (DMSO) δH 13.55 (brs, 1H, NH), 12.25 (s, 1H, NH), 8.62 (s, 1H, Ar-CH), 8.45 (d, 2H, J: 9, Ar-CH), 8.37 (d, 2H, J: 9; Ar-CH), 8.15 (s, 1H, thiazole), 7.52 (d, 1H, J: 8.5, Ar-CH), 7.2 (d, 1H, J: 8.5, Ar-CH)	
**5a**	4-C_6_H_5_	69.5	62.58; 3.42; 15.86 62.57; 3.41; 15.85	^1^H-NMR (DMSO) δH 13.55 (brs, 1H, NH), 12.25 (s, 1H, NH), 8.6 (s, 1H, Ar-CH), 7.77 (s, 1H, thiazole), 7.54 (m, 4H, Ar-CH), 7.48 (m, 3H, Ar-CH), 7.32 (d, 2H, Ar-CH), 7.22 (t, 1H, Ar-CH), 7.21 (d, 1H, J: 8.5, Ar-CH)	
**6a**	4-CN	69.3	55.38; 2.58; 21.53 55.36; 2.57; 21.54	^1^H-NMR (DMSO) δH 13.55 (brs, 1H, NH), 12.22 (s, 1H, NH), 8.06 (s, 1H, Ar-CH), 8.22 (d, 2H, J: 9, Ar-CH), 8.00 (d, 2H, J: 9, Ar-CH), 8.05 (s, 1H, thiazole), 7.48 (d, 1H, J: 8.5, Ar-CH), 7.11 (d, 1H, J: 8.5, Ar-CH)	
**7a**	2,4-F	66	50.87; 2.26; 17.45 50.88; 2.26; 17.44	^1^H-NMR (DMSO) δH 13.55 (brs, 1H, NH), 12.22 (s, 1H, NH), 8.6 (s, 1H, Ar-CH), 8.28 (d, 1H, J: 8.5, Ar-CH), 7.6 (s, 1H, thiazole), 7.48 (d, 1H, J: 8.5, Ar-CH), 7.45 (s, 1H, Ar-CH), 7.35 (d, 1H, J: 8.5, Ar-CH), 7.11 (d, 1H, J: 8.5, Ar-CH)	
**8a**	3-NO_2_	43	49.76; 2.46; 20.48 49.77; 2.45; 20.47	^1^H-NMR (DMSO) δH 13.55 (brs, 1H, NH), 12.22 (s, 1H, NH), 8.6 (s, 1H, Ar-CH), 8.56 (d, 1H, J: 8, Ar-CH), 8.36 (d, 1H, J: 8, Ar-CH), 8.1 (s, 1H, thiazole), 7.90 (t, 1H, J: 8, Ar-CH), 7.48 (m, 2H), 7.11 (d, 1H, J: 8.5, Ar-CH)	
**9a**	3,4-Cl	50	47.02; 2.09; 16.13 47.01; 2.09; 18.45	^1^H-NMR (DMSO) δH 13.55 (brs, 1H, NH), 12.22 (s, 1H, NH), 8.6 (s, 1H, Ar-CH), 8.4 (s, 1H, Ar-CH), 8.15 (d, 1H, J: 8, Ar-CH), 7.72 (s, 1H, thiazole), 7.67 (d, 1H, J: 8, Ar-CH), 7.48 (d, 1H, J: 8.5, Ar-CH), 7.11 (d, 1H, J: 8.5, Ar-CH)	
**10a**	4-CH_3_	69	56.98; 3.45; 18.46 56.99; 3.45; 18.45	^1^H-NMR (DMSO) δH 13.55 (brs, 1H, NH), 12.22 (s, 1H, NH), 8.6 (s, 1H, Ar-CH), 7.94 (d, 2H, *J*: 9, Ar-CH), 7.76 (s, 1H, thiazole), 7.48 (d, 1H, J: 8.5, Ar-CH), 7.37 (d, 2H, J: 9, Ar-CH), 7.11 (d, 1H, J: 8.5, Ar-CH), 2.35 (s, 3H, CH_3_)	
**11a**	4-OCH_3_	72	54.68; 3.31; 17.71 54.69; 3.31; 17.70	^1^H-NMR (DMSO) δH 13.55 (brs, 1H, NH), 12.22 (s, 1H, NH), 8.6 (s, 1H, Ar-CH), 8.03 (d, 2H, J: 8.5, Ar-CH), 7.66 (s, 1H, thiazole), 7.48 (d, 1H, J: 8.5, Ar-CH), 7.14 (d, 2H, J: 8.5, Ar-CH), 7.11 (d, 1H, J: 8.5, Ar-CH), 3.85 (s, 3H, OCH_3_)	
**12a**	H	63	55.88; 3.03; 19.17 55.87; 3.02; 19.16	^1^H-NMR (DMSO) δH 13.55 (brs, 1H, NH), 12.22 (s, 1H, NH), 8.6 (s, 1H, Ar-CH), 8.1 (d, 2H, J: 9, Ar-CH), 7.84 (s, 1H, thiazole), 7.57 (d, 2H, J: 9, Ar-CH), 7.48 (d, 1H, J: 8.5, Ar-CH), 7.47 (t, 1H, J: 8.5, Ar-CH), 7.11 (d, 1H, J: 8.5, Ar-CH)	

### General procedure for the synthesis of compounds 1b–12b

According to the above-described procedure, equimolar amounts of 5-chloroisatin and hydrazinecarbothioamide in 2-propanol were refluxed in ethanol in the presence of catalytic amounts of acetic acid to give 2-(5-chloro-2-oxoindolin-3-ylidene)hydrazine-1-carbothioamide with a yield of 94%; Rf = 0.31 (ethyl-acetate/hexane = 1/1); MS (ESI+): 255.01 ([M+H]^+^). The purified carbothioamide was then reacted accordingly to the previously described method to synthesize compounds 1b–12b (**Table 2**).

**Table 2:** Analytical data of derivatives 1b–12b.

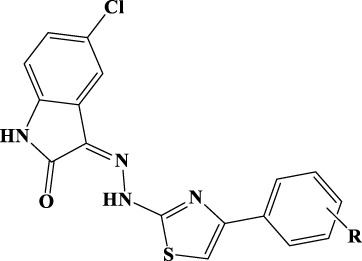				
Comp.	R	% Yield	CHN	^1^H-NMR
			Calc./found	
**1b**	4-Cl	61	52.45; 2.59; 14.39 52.46; 2.58; 14.38	^1^H-NMR (600 MHz, DMSO) δH 13.35 (bs, 1H, NH), 11.37 (s, 1H, NH), 7.93 (d, 2H, J: 8.58, CH-Ar), 7.84 (s, 1H, thiazole), 7.54 (d, 1H, J: 2.16, CH-5Cl isa), 7.49 (d, 2H, J: 8.58, CH-Ar), 7.39 (dd, 1H, J: 2.16–8.34), 6.99 (d, 1H, J: 8.28, CH-5Cl isa)
**2b**	4-F	57	54.77; 2.70; 15.03 54.76; 2.69; 15.02	^1^H-NMR (600 MHz, DMSO) δH 13.32 (bs, 1H, NH), 11.40 (s, 1H, NH), 7.95 (m, 2H, CH-Ar), 7.65 (s, 1H, thiazole), 7.52 (d, 1H, J: 1.32, CH-5Cl isa), 7.38 (dd, 1H, J: 1.8–8.28, CH-Cl isa), 7.26 (m, 2H, CH-Ar), 6.98 (d, 1H, J: 8.34, CH-5Cl isa)
**3b**	4-Br	62	47.08; 2.32; 12.92 47.07; 2.31; 12.91	^1^H-NMR (600 MHz, DMSO) δH 13.34 (s, 1H, NH), 11.36 (s, 1H, NH), 7.87 (d, 2H, J: 8.22, CH-Ar), 7.75 (s, 1H, thiazole), 7.63 (d, 2H, J: 8.28, CH-Ar), 7.54 (s, 1H, CH-Cl isa), 7.39 (d, 1H, J: 7.98, CH-5Cl isa), 6.99 (d, 1H, J: 8.4, CH-5Cl isa)
**4b**	4-NO_2_	68	51.07; 2.52; 17.52 51.05; 2.52; 17.51	^1^H-NMR (600 MHz, DMSO) δH 13.38 (bs, 1H, NH), 11.38 (s, 1H, NH), 8.29 (d, 2H, J: 8.94, CH-Ar), 8.18 (d, 2H, J: 8.88, CH-Ar), 8.05 (s, 1H, thiazole), 7.55 (d, 1H, J: 1.98, CH-5Cl isa), 7.4 (dd, 1H, J: 2.1–8.28, CH-5Cl isa), 6.99 (d, 1H, J: 8.28, CH-5Cl isa)
**5b**	4-C_6_H_5_	69	64.11; 3.51; 13.00 64.10; 3.50; 12.99	^1^H-NMR (600 MHz, DMSO) δH 13.37 (bs, 1H, NH), 11.37 (s, 1H, NH), 8.00 (d, 2H, J: 8.46 CH-Ar), 7.73–7.77 (m,5H), 7.56 (s, 1H, J: 1.98, CH-5Cl isa), 7.52–7.48 (m, 2H), 7.43–7.38 (m, 2H), 6.99 (d, 1H, J: 8.34, CH-5Cl isa)
**6b**	4-CN	69.5	56.92; 2.65; 18.44 56.90; 2.64; 18.45	^1^H-NMR (600 MHz, DMSO) δH 13.36 (s, 1H, NH), 11.38 (s, 1H, NH), 8.10 (d, 2H, J: 7.98, CH-Ar), 7.97 (s, 1H, CH-5Cl isa), 8.90 (d, 2H, J: 7.74, CH-Ar), 7.55 (s, 1H, thiazole), 7.40 (d, 1H, J: 7.8, CH-5Cl isa), 6.99 (d, 1H, J: 8.22, CH-5Cl isa)
**7b**	2,4-F	67	52.25; 2.32; 14.34 52.23; 2.31; 14.33	^1^H-NMR (600 MHz, DMSO) δH 13.34 (s, 1H, NH), 11.37 (s, 1H, NH), 7.93 (m, 1H), 7.84 (m, 2H), 7.41–7.37 (m, 2H), 7.21–7.18 (m, 1H), 6.99 (d, 1H, J: 8.34, CH-5Cl isa)
**8b**	3-NO_2_	50	51.07; 2.52; 17.52 51.08; 2.51; 17.51	^1^H-NMR (600 MHz, DMSO) δH 13.38 (s, 1H, NH), 11.38 (s, 1H, NH), 8.69 (s,1H, CH-Ar), 8.37 (d, 1H, J: 7.8, CH-Ar), 8.20 (m, 1H, CH-Ar), 8.0 1 (s, 1H, thiazole), 7.75 (t, 1H, J: 7.8, CH-Ar), 7.55 (d, 1H, J: 1.86, CH-5Cl isa), 7.39 (dd, 1H, J: 2.04–8.34, CH-5Cl isa), 6.99 (d, 1H, J: 8.28, CH-5Cl isa)
**9b**	3,4-Cl	51	48.19; 2.14; 13.22 48.20; 2.13; 13.21	^1^H-NMR (600 MHz, DMSO) δH 13.35 (brs, 1H, NH), 11.38 (s, 1H, NH), 8.16 (m, 1H, CH-Ar), 7.92–7.89 (m, 2H), 7.70 (d, 1H, J:8.28, CH-Ar), 7.75 (s, 1H, CH-5Cl isa), 7.39 (dd, 1H, J: 1.92 8.04, CH-5Cl isa), 6.99 (d, 1H, J: 8.62, CH-5Cl isa)
**10b**	4-CH_3_	68	58.61; 3.55; 15.19 58.62; 3.54; 15.18	^1^H-NMR (600 MHz, DMSO) δH 13.34 (bs, 1H, NH), 11.36 (s, 1H, NH), 7.80 (d, 2H, J: 8.04, CH-Ar), 7.60 (s, 1H, thiazole), 7.54 (m, 1H, CH-5Cl isa), 7.39 (dd, 1H, J: 2.04 8.34, CH-5Cl isa), 7.25 (d, 2H, J: 8.04, CH-Ar), 6.99 (d, 1H, J: 8.34, CH-5Cl isa) 2.37 (s, 3H, CH3-Ar)
**11b**	4-OCH_3_	71	56.18; 3.40; 14.56 56.19, 3.39; 14.55	^1^H-NMR (600 MHz, DMSO) δH 13.34 (bs, 1H, NH), 11.36 (s, 1H, NH), 7.84 (d, 2H, J: 7.89, CH-Ar), 7.54 (s, 1H) 7.51 (s, 1H), 7.40 (m, 1H, CH-5Cl isa), 6.99 (d, 2H, J: 8.34, CH-Ar) 6.85 (m, 1H, CH-5Cl isa), 3.83 (s, 3H, OCH3-Ar)
**12b**	H	61	57.55; 3.12; 15.79 57.54; 3.11; 15.78	^1^H-NMR (600 MHz, DMSO) δH 13.35 (bs, 1H, NH), 11.37 (s, 1H, NH), 7.91 (d, 2H, J: 7.68, CH-Ar), 7.68 (s, 1H) 7.55 (s, 1H), 7.45 (t, 2H, J: 7.56, CH-Ar), 7.39 (m, 1H, CH-5Cl isa) 7.35 (t, 1H, J: 7.56, CH-Ar), 6.99 (d, 1H, J: 7.98, CH-5Cl isa)

### General procedure for the synthesis of compounds 1b–12b

To a solution of hydrazine hydrate in ethanol, an equimolar amount of methyl isothiocyanate was added dropwise. The solution was stirred overnight at rt. A white precipitate was filtered and crystallized from ethanol to give *N*-methylhydrazinecarbothioamide (Mp. 135°C–137°C) in 89% yield. The obtained carbothioamide was then reacted with 5-nitroisatin in 2-propanol. The reaction was stirred under reflux overnight to give *N*-methyl-2-(5-nitro-2-oxoindolin-3-ylidene)hydrazine-1-carbothioamide. Yield: 96.57%; MS (ESI+): 280.05 ([M+H]^+^). The obtained oxoindolcarbothioamide was then reacted with the appropriate α-halogen aryl-ketone in refluxing 2-propanol to give the desired 3-[(3-methyl-4-arylthiazol-2-ylidene)hydrazineylidene]-5-nitroindolin-2-ones 1c–12c (**Table 3**).

**Table 3:** Analytical data of derivatives 1c–12c.

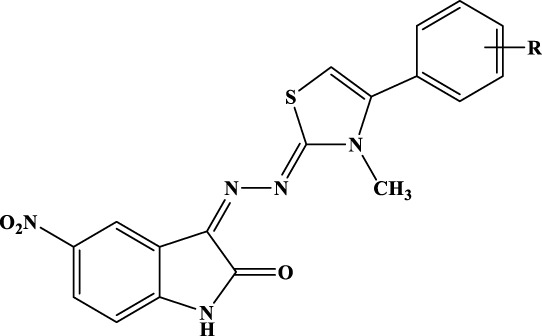				
Comp.	R	% Yield	CHN	^1^H-NMR
			Calc. found	
**1c**	4-Cl	34	52.24; 2.92; 16.92 52.26; 2.91; 16.93	^1^H-NMR (DMSO) δ (ppm): 11.18 (s, 1H), 9.01 (d, 1H), 8.20 (dd, 1H), 7.66 (d, 2H), 7.63 (d, 2H), 7.03 (m, 2H), 3.65 (s, 3H)
**2c**	4-F	53	54.40; 3.04; 17.62 54.38; 2.95; 17.65	^1^H-NMR (DMSO) δ (ppm): 11.18 (s, 1H), 9.01 (d, 1H), 8.20 (dd, 1H), 7.69 (dd, 2H), 7.41 (m, 2H), 7.03 (d, 1H), 6.98 (s, 1H), 3.63 (s, 3H)
**3c**	4-Br	50	47.17; 2.64; 15.28 47.21; 2.61; 15.32	^1^H-NMR (DMSO) δ (ppm): 11.19 (s, 1H), 9.02 (d, 1H), 8.20 (dd, 1H), 7.76 (d, 2H), 7.59 (d, 2H), 7.04 (m, 2H), 3.65 (s, 3H)
**4c**	4-NO_2_	67	50.94; 2.85; 19.80 50.87; 2.90; 19.73	^1^H-NMR (DMSO) δ (ppm): 11.22 (s, 1H), 9.04 (d, 1H), 8.38 (m, 2H), 8.22 (dd, 1H), 7.94 (d, 2H), 7.20 (s, 1H); 7.02 (s, 1H), 3.69 (s, 3H)
**5c**	4-C_6_H_5_	48	63.29; 3.76; 15.38 63.31; 3.75; 15.37	^1^H-NMR (DMSO) δ (ppm): 11.20 (s, 1H), 9.04 (d, 1H), 8.21 (dd, 1H), 7.86 (d, 2H), 7.77 (d, 2H), 7.73 (d, 2H), 7.52 (t, 2H), 7.43 (t, 1H), 7.05 (d, 2H), 3.72 (s, 3H)
**6c**	4-CN	798	56.43; 2.99; 20.78 56.45; 2.98; 20.79	^1^H-NMR (DMSO) δ (ppm): 11.19 (s, 1H), 9.01 (d, 1H), 8.39 (ddd, 1H), 8.20 (d, 1H), 8.11 (m, 1H), 8.04 (m, 1H), 7.85 (m, 1H), 7.17 (s, 1H), 7.03 (d, 1H), 3.66 (s, 3H)
**7c**	2,4-F	54	52.05; 2.67; 16.86 52.02; 2.66; 16.87	^1^H-NMR (DMSO) δ (ppm): 11.63 (s, 1H), 9.17 (d, 1H), 8.68 (m, 1H), 7.93 (m, 1H), 7.81 (m, 1H), 7.02 (s, 1H), 6.87 (d, 1H), 6.81 (d, 1H), 3.67 (s, 3H)
**8c**	3-NO_2_	41	50.94; 2.85; 19.80 50.95; 2.84; 19.81	^1^H-NMR (DMSO) δ (ppm): 11.21 (s, 1H), 9.04 (s, 1H), 8.47 (s, 1H), 8.39 (dd, 1H), 8.21 (d, 1H), 8.11 (d, 1H), 7.86 (m, 1H), 7.18 (s, 1H), 7.05 (m, 1H), 3.67 (s, 3H)
**9c**	3,4-Cl	64,	48.23; 2.47; 15.62; 48.15; 2.46; 15.61	^1^H-NMR (DMSO) δ (ppm): 11.19 (s, 1H), 9.01 (d, 1H), 8.20 (dd, 1H), 7.95 (d, 1H), 7.83 (d, 1H), 7.64 (dd, 1H), 7.10 (s, 1H), 7.04 (s, 1H), 3.66 (s, 3H)
**10c**	4-CH_3_	44	58.01; 3.84; 17.80 58.03; 3.85; 17.79	^1^H-NMR (DMSO) δ (ppm): 11.15 (s, 1H), 8.96 (d, 1H), 8.16 (dd, 1H), 7.50 (d, 2H), 7.37 (d, 2H), 7.00 (d, 1H), 6.91 (s, 1H), 3.63 (s, 3H), 2.40 (s, 3H)
**11c**	4-OCH_3_	37	55.74; 3.69; 17.11 85.75; 3.68; 17.12	^1^H-NMR (DMSO) δ (ppm): 11.17 (s, 1H), 9.00 (d, 1H), 8.19 (dd, 1H), 7.55 (d, 2H), 7.10 (d, 2H), 7.03 (d, 1H), 6.89 (s, 1H), 3.84 (s, 3H), 3.64 (s, 3H)
**12c**	H	58	56.98; 3.45; 18.46 57.00; 3.46; 18.50	^1^H-NMR (DMSO) δ (ppm): 11.16 (s, 1H), 8.98 (d, 1H), 8.18 (dd, 1H), 7.63 (m, 2H), 7.56 (m, 3H), 7.01 (d, 1H), 6.97 (s, 1H), 3.65 (s, 3H)

### Expression and purification of recombinant HIV-1 RT

#### HIV-1 RT group M subtype B

*Escherichia coli* strain M15 containing the p6HRT-prot vector (provided by Stuart Le Grice laboratory (NCI Frederick)) was grown to an optical density at 600 nm of 0.7 and induced with 1.7 mM of isopropyl β-d-1-thiogalactopyranoside (IPTG) for 4 h. Protein purification was carried out with a BioLogic LP system (Bio-Rad, Hercules, CA, USA) using a combination of immobilized metal affinity chromatography (IMAC) and ion exchange chromatography. Cell pellets were resuspended in lysis buffer (50 mM of sodium phosphate buffer, pH 7.8, containing 0.5 mg/mL of lysozyme), incubated on ice for 20 min, and, after the addition of NaCl to a final concentration of 0.3 M, were sonicated and centrifuged at 30,000 ×*g* for 1 h. The supernatant was loaded onto a Ni^2+^-NTA-Sepharose column pre-equilibrated with loading buffer (50 mM of sodium phosphate buffer, pH 7.8, containing 0.3 M of NaCl, 10% glycerol, and 10 mM of imidazole) and washed thoroughly with wash buffer (50 mM of sodium phosphate buffer, pH 6.0, containing 0.3 M of NaCl, 10% glycerol, and 80 mM of imidazole). RT was eluted with an imidazole gradient in the wash buffer (0–0.5 M). Fractions were collected, and protein purity was checked by sodium dodecyl sulfate–polyacrylamide gel electrophoresis (SDS-PAGE) and found to be higher than 90%. The 1:1 ratio between the p66/p51 subunits was also verified. Enzyme-containing fractions were pooled and diluted 1:1 with 50 mM of sodium phosphate buffer, pH 7.0, containing 10% glycerol, and then loaded into a Hi-trap heparin HP GE (Healthcare LifeScience) pre-equilibrated with 10 column volumes of loading buffer (50 mM of sodium phosphate buffer, pH 7.0, containing 10% glycerol, and 150 mM of NaCl). The column was then washed with loading buffer, and the RT was eluted with Elute Buffer 2 (50 mM of sodium phosphate, pH 7.0, 10% glycerol, 1 M of NaCl). Fractions were collected, and the protein was dialyzed and stored in a buffer containing 50 mM of Tris-HCl, pH 7.0, 25 mM of NaCl, 1 mM of EDTA, and 50% glycerol. Catalytic activities and protein concentrations were determined. Enzyme-containing fractions were pooled, and aliquots were stored at −80°C.

#### Site-directed mutagenesis

K103N, Y181C, and Y188L mutations were introduced into the p6HRT-prot vector using the QuikChange protocol (Agilent Technologies, Santa Clara, CA, USA). Enzymes were expressed and purified as the wt-HIV-1 RT.

### HIV-1 DNA polymerase-independent RNase H activity determination

HIV RT-associated RNase H activity was measured as described (Corona et al., 2016b) in a 100-µl reaction volume containing 50 mM of Tris-HCl buffer, pH 7.8, 6 mM of MgCl_2_, 1 mM of dithiothreitol (DTT), 80 mM of KCl, 0.25 µM of hybrid RNA/DNA 5′-GAUCUGAGCCUGGGAGCU-Fluorescin-3′ (high-performance liquid chromatography (HPLC), dry, QC: Mass Check) (available from Metabion) 5′-Dabcyl-AGCTCCCAGGCTCAGATC-3′ (HPLC, dry, QC: Mass Check), increasing concentrations of inhibitors, whose dilution were made in water, and different amounts of enzymes according to a linear range of the dose–response curve. The reaction mixture was incubated for 1 h at 37°C and stopped by the addition of EDTA, and products were measured with a multilabel counter plate reader Victor 3 equipped with filters for 490/528 nm.

### HIV-1 RNA-dependent DNA polymerase activity determination

RDDP activity was measured as previously reported in 25-µl volume containing 60 mM of Tris‐HCl buffer, pH 8.1, 8 mM of MgCl_2_, 60 mM of KCl, 13 mM of DTT, 2.5 µM of poly(A)-oligo(dT), 100 µM of dTTP, increasing concentrations of inhibitors, whose dilution were made in water, and different amounts of enzymes according to a linear range of the dose–response curve. The reaction mixture was incubated for 30 min at 37°C and then stopped by the addition of EDTA. Reaction products were detected by pico green addition and measured with a multilabel counter plate reader Victor 3, equipped with filters for 502/523 nm.

### Preparation of recombinant full-length IN and LEDGF proteins

Proteins were expressed in *E. coli* BL21(DE3). Briefly, His-IN was purified by loading the precipitate of cell lysate onto a Ni-Sepharose column and eluting with a decreasing imidazole gradient (0–500 mM) in a HEPES buffer (50 mM, pH 7.5) containing NaCl (1 M), CHAPS (7.5 mM), and β-mercaptoethanol (2 mM). FLAG-IN was purified by loading the precipitate of cell lysate onto a phenyl-sepharose and ammonium sulfate gradient (0–800 mM) in a HEPES (pH 7.5, 50 mM) buffer containing NaCl (200 mM), CHAPS (7.5 mM), and β-mercaptoethanol (2 mM). Peak fractions were pooled, loaded onto a heparin column, and eluted with an increasing NaCl gradient (200 mM to 1 M) in a HEPES (pH 7.5, 50 mM) buffer containing CHAPS (7.5 mm) and β-mercaptoethanol (2 mM). Fractions containing integrase were pooled and stored in glycerol (10%) at −80°C.

### HTRF LEDGF-dependent and LEDGF-independent assay

The IN LEDGF/p75-dependent assay allows measurement of the inhibition of 3′-processing and strand-transfer IN reactions in the presence of recombinant LEDGF/p75 protein. Briefly, IN (50 nM) was preincubated with increasing concentrations of compounds for 1 h at room temperature in a reaction buffer containing HEPES (20 mM, pH 7.5), DTT (1 mM), glycerol (1%), MgCl_2_ (20 mM), Brij-35 (0.05%), and bovine serum albumin (BSA) (0.1 mg/ml). To this mixture, DNA donor substrate, DNA acceptor substrate, and LEDGF/p75 protein (50 nm, or with the omission of LEDGF/p75 protein) were added, and incubation was performed at 378°C for 90 min. After the incubation, Europium/streptavidin reader was used with lex = 314 nm and lem = 668 and 620 nm for the acceptor and donor substrates, respectively.

### HTRF-based integrase-LEDGF interaction assay

This assay was carried out as described (Tsiang et al., 2009; Kessl et al., 2012; Martini et al., 2017). Briefly, His-IN was preincubated with different concentrations of a compound in a buffer containing NaCl (150 mm), MgCl_2_ (2 mM), Nonidet P-40 (0.1%), BSA (1 mg/ml), and Tris (pH 7.4, 25 mm) for 30 min at room temperature. Then, FLAG-LEDGF was added to the reaction mixture, and a mixture of anti-His6-XL665 and anti-FLAG-EuCryptate antibodies was added. After 4 h at 48°C, the homogeneous time-resolved fluorescence (HTRF) signal was recorded with a PerkinElmer Victor 3 plate reader with the use of 314 nm for the excitation wavelength and 668 and 620 nm for the wavelengths of the acceptor and donor emission, respectively. The HTRF signal is defined as the emission ratio 665/620 nm multiplied by 10,000.

### HTRF-based IN subunit exchange assay

His and FLAG-tagged INs were mixed in Tris (pH 7.4, 25 mm) buffer containing NaCl (150 mm), MgCl_2_ (2 mM), Nonidet P-40 (0.1%), and BSA (1 mg/ml). Test compounds were then added to the mixture, and incubation was carried out for 2.5 h at room temperature. A mixture of anti-His6-XL665 and anti-FLAG-EuCryptate antibodies was then added, and incubation was carried out at room temperature (Corona et al., 2016a).

### HIV-1 replication inhibition

HIV-1 replication was carried out in MT4 cells as previously reported (Corona et al., 2016a) using a molecular clone derived from the pNL4-3 (Thierry et al., 2015). A total of 120 ng of p24^gag^ antigen per 10^6^ cells, corresponding to a multiplicity of infection (m.o.i.) of 0.3, was used for infection. Cells were treated in the presence of RAL (500 nM) (Selleckchem, Houston, TX, USA) or EFV (100 nM) (Sigma-Aldrich, St. Louis, MO, USA) as the positive control. Flow cytometry analysis was performed on a FACSCalibur flow cytometer (BD Biosciences, San Jose, CA, USA), and the results were analyzed using the ImageQuant software.

### Quantification of viral DNA genomes

Quantitative PCR (qPCR) was performed as described previously (Munir et al., 2013). Briefly, MT4 cells were infected using target compounds and RAL at 500 nM and EFV at 100 nM as controls. Cells were washed in phosphate-buffered saline (PBS), and dry cell pellets were frozen at −80°C until use. DNA from samples was purified with QIAamp DNA Blood mini kit (Qiagen, Valencia, CA, USA) according to the manufacturer’s instructions. Quantification of total viral DNAs, 2-LTR circular viral DNA (2-LTRc), and integrated DNAs was performed on a LightCycler instrument (Roche Diagnostics, Basel, Switzerland) using the second-derivative-maximum method provided by the LightCycler quantification software, version 3.5 (Roche Diagnostics). Amplification of the β-globin gene (two copies per diploid cell) was performed to normalize the results using the commercially available materials (Control kit DNA; Roche Diagnostics). The quantification of the results for 2-LTRc and total HIV-1 DNA was expressed as copy numbers per µg DNA. Integrated HIV-1 DNA was expressed as a percentage of wt control. Data were represented by mean and standard deviation of two independent experiments; p-values were calculated between the infected and infected-treated conditions by unpaired, two-tailed t-tests using GraphPad Prism Version 9.5.1 software (GraphPad Software, Inc., San Diego, CA, USA). Figures were drawn with GraphPad Prism Version 9.5.1.

### Molecular modeling

#### Ligand preparation

A theoretical 3D model of compound **10a** was built by means of Maestro GUI. The lowest energy conformer was considered for the following studies. This was obtained from the starting conformations through a conformational search by means of MacroModel version 7.2 (Mohamadi et al., 1990), considering MMFFs (Halgren, 1996) as a force field and solvent effects by adopting the implicit solvation model generalized Born/surface area (GB/SA) water (Still et al., 1990). The simulations were performed allowing 1,000 steps of Monte Carlo analysis with the Polak–Ribière conjugate gradient (PRCG) method and a convergence criterion of 0.05 kcal/(molÅ).

#### RT protein preparation

The coordinates for RT enzymes were downloaded from the RCSB Protein Data Bank (Berman et al., 2000) [PDB codes 1vrt (Ren et al., 1995), 2zd1 (Das et al., 2008), 1ep4 (Ren et al., 2000), 3qo9 (Das et al., 2011), 1rti (Ren et al., 1995), 1tv6 (Pata et al., 2004), and 3lp2 (Himmel et al., 2009)]. The proteins were prepared by using the Maestro Protein Preparation Wizard protocol.

#### Docking experiments

**Figure 2:**
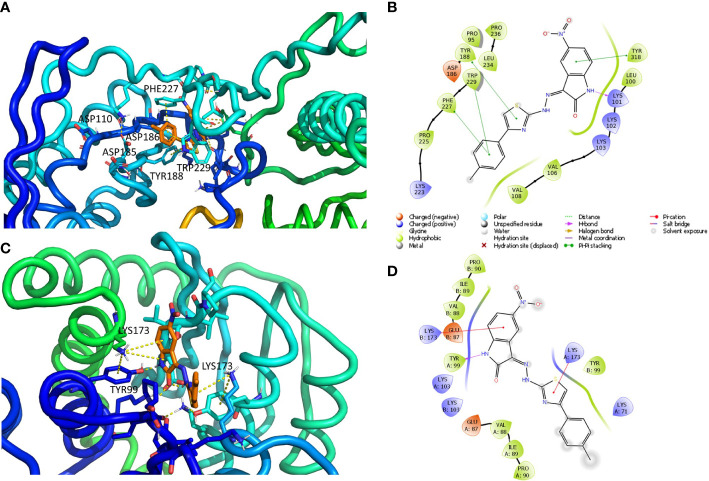
Putative binding mode obtained through docking experiments considering the two targets, RT (pdb code 1tv6) and IN (pdb code 2b4j) of HIV-1, and the most promising compound synthesized, 10a. **(A)** RT–polymerase catalytic site (Asp110, Asp185, and Asp186), and the adjacent NNRTI pocket is represented as cartoons and the compound in orange sticks. **(B)** Corresponding 2D image of the compounds and the enzyme interacting residues. **(C)** IN dimer interface is represented as cartoons and the compound in orange. **(D)** Corresponding 2D image of the compound and the enzyme interacting residues. RT, reverse transcriptase; IN, integrase; NNRTI, non-nucleoside reverse transcriptase inhibitor.

Default settings were applied. The enzyme was divided into boxes of the same size (46 × 46 × 46 Å) covering overall the whole p66 subunit. The docking grids were defined by centering on W229 and Q500. Ensemble docking experiments were carried out applying QMPL considering the XP scoring function. The best solution was analyzed and reported in **Figures 2A, B**.

#### IN protein preparation

The crystal structure of HIV-1 IN protein catalytic core domain complexed with the IN binding domain of LEDGF/p75 (PDB ID: 2b4j) (Cherepanov et al., 2005) was used for the docking. The LEDGF/p75 protein and water molecules were removed from the crystal structure, and the default protein preparation wizard protocol was applied.

#### Docking experiments

The docking grid (60 × 60 × 60 Å) was defined by centering it and including the whole dimer enzyme. Docking was carried out by applying QMPL with XP. The best solution was analyzed and reported in **Figures 2C, D**.

The resulting docking complexes were considered for the binding mode graphical analysis with Maestro and PyMOL (PyMOL Molecular Graphics System Schrödinger, LLC.).

## Results and discussion

A small library of 5-nitro- and 5-chloro-indolinone derivatives, namely, compounds 1a–12a, 1b–12b, and 1c-12c, was designed and synthesized to explore their ability to inhibit both associated functions of HIV RT. According to a previously described procedure (Meleddu et al., 2015; Meleddu et al., 2017), the synthesis consists of a multi-step approach, starting from the reaction of the 5-nitro or 5-chloro-substituted indolinone with hydrazinecarbothioamide, as in the case of compounds 1a–12a and 1b–12b, and *N*-methylhydrazine carbothioamide, as in the case of compounds 1c–12c, to give the intermediate thiosemicarbazones and methyl-thiosemicarbazones, which were further reacted with the appropriate alpha halogen-aryl ketones to furnish the desired compounds. Therefore, three families of compounds were synthesized, namely, two series of hydrazothiazoles 1a–12a and 1b–12b and one series of hydrazo-*N*-methyl-dihydrothiazoles, namely, compounds 1c–12c. All compounds were purified by either crystallization or column chromatography and submitted to biological testing.

When tested on the two enzymatic RT functions, all the new compounds exhibited dual activity with very few exceptions, as for compounds 6c and 8c, which were active only toward the RDDP function (**Table 4**). However, two compounds of the *N*-methyl-dihydrothiazole series, 4c and 9c, were the most potent inhibitors of the RNase H function, with an IC_50_ value of 1.9 μM. Compound 4c was also the most active derivative toward the RDDP function, with an IC_50_ value of 6.0 μM. According to the enzymatic evaluation results, the *N*-methyl-dihydrothiazole series could be considered the most promising one among the three tested families.

**Table 4:** Effect of 1–12a–c derivative compounds on HIV-1 RT-associated RNase H and RDDP activities.

		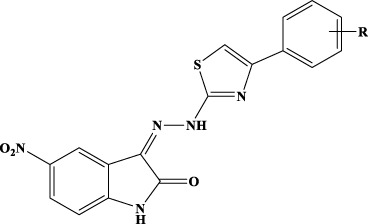		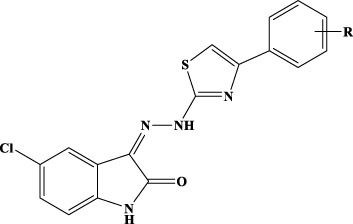		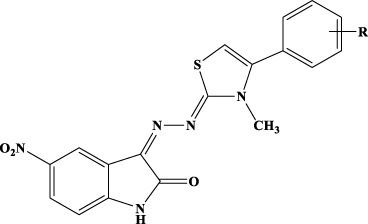	
		Compounds 1a–12a		Compounds 1b–12b		Compounds 1c–12c	
Comp.	R-Ph	RNase H IC_50_ (µM)^a^	RDDP IC_50_ (µM)^b^	RNase H IC_50_ (µM)^a^	RDDP IC_50_ (µM^b^	RNase H IC_50_ (µM)^a^	RDDP IC_50_ (µM)^b^
**1**	**4Cl**	4.6 ± 1.4	10 ± 0.5	12 ± 2.3	30 ± 7.5	8.2 ± 1.0	24 ± 3
**2**	**4F**	5.1 ± 2.0	9.5 ± 0.5	4.5 ± 0.2	7.5 ± 1.5	5.6 ± 0.6	21 ± 1
**3**	**4Br**	4.3 ± 0.7	8.5 ± 0.5	4.4 ± 0.1	8.3 ± 0.6	7.7 ± 1.2	19.5 ± 3.3
**4**	**4NO_2_**	7.6 ± 1.4	21 ± 3	15.3 ± 1.5	14.9 ± 2.7	1.9 ± 0.2	6.0 ± 0.5
**5**	**4C_6_H_5_**	7.1 ± 0.1	12 ± 3.0	13.2 ± 1.6	38 ± 5	6.1 ± 1.1	11.4 ± 2.2
**6**	**4CN**	6.0 ± 0.1	9.3 ± 0.3	7.8 ± 0.1	34 ± 6	100 ± 5	12.1 ± 1.6
**7**	**2-4F**	6.0 ± 0.1	11.5 ± 0.5	6 ± 1.5	10 ± 1.0	ND	ND
**8**	**2NO_2_**	6.5 ± 0.5	18.5 ± 0.5	22 ± 1	72 ± 13	100 ± 20	8.7 ± 1.5
**9**	**3-4Cl**	6.5 ± 0.5	11.5 ± 0.5	8.8 ± 1.3	24 ± 3	1.9 ± 0.7	15.1 ± 0.5
**10**	**4CH_3_**	8.5 ± 0.5	18 ± 1.5	10 ± 1.4	18 ± 0.7	6.8 ± 1.4	16.9 ± 2.3
**11**	**4OCH_3_**	8.5 ± 0.6	14.5 ± 0.5	7 ± 1.0	16.2 ± 2.8	4 ± 1.4	11.2 ± 2.2
**12**	**H**	6.0 ± 1.0	13 ± 2	5.4 ± 0.6	9.4 ± 1.1	3.6 ± 0.8	8.1 ± 0.1

a Concentration required to inhibit HIV-1 RT-associated RNase H activity by 50% obtained by three independent experiments (reported as average ± standard deviation).

b Concentration required to inhibit HIV-1 RT-associated RDDP activity by 50% obtained by three independent experiments (reported as average ± standard deviation).

c Fold of increase with respect to wt RT.

Furthermore, all compounds with an IC_50_ value lower than 20 μM against both enzymatic functions were tested for their ability to inhibit viral replication in cell cultures (**Table 5**). Unfortunately, although interesting in the enzymatic assays, *N*-methyl-dihydrothiazole showed greater cytotoxicity when compared to the hydrazothiazole congeners. Among the hydrazothiazole derivatives, four compounds were found active in the replication assay, namely, compounds 1a, 3a, 10a, and 9b, with EC_50_ values of 25, 32, 15.8, and 13.2 μM, respectively. Interestingly, none of these four derivatives exhibited toxicity up to 100 μM (the highest concentration tested) against MT4 cells. Compounds 10a and 9b were the most potent and promising derivatives. However, considering that nitro-indole derivatives were generally better performing in the cell-based replication assays, compound 10a was chosen for further investigation on the mechanism of action, and 9b was flanked to the comparative study as a selective inhibitor for RT.

**Table 5:** Biological effects of –12a–c derivatives on HIV-1 replication.

		Compounds 1a–12a		Compounds 1b–12b			Compounds 1c–12c			
Cp	R-Ph	HIV-1	MT4	HIV-1		MT4	HIV-1		MT4	
		EC_50_ (µM)^a^	CC_50_ (µM) ^b^	EC_50_ (µM) ^a^		CC_50_ (µM) ^b^	EC_50_ (µM) ^a^		CC_50_ (µM)^b^	
1	**4Cl**	25 ± 2.1	>100	>100		>100	>42		42 ± 8	
2	**4F**	>100	>100	>56		56 ± 6	>7		7 ± 1.6	
3	**4Br**	32 ± 3.2	>100	>95		95 ± 14	>25		25 ± 3.1	
4	**4NO_2_**	>100	>100	>50		50 ± 12	>5.5		5.5 ± 0.9	
5	**4C_6_H_6_**	>100	>100	ND		ND	>7		7 ± 1.4	
6	**4CN**	>100	>100	>95		95 ± 5	ND		ND	
9	**3,4Cl**	>100	>100	13.2 ± 3.7		>100	>5		5 ± 1.3	
10	**4CH_3_**	15.8 ± 1.8	>100	>100		>100	>10		10 ± 2.7	
11	**4OCH_3_**	>100	>100	>70		70 ± 9	>2		2 ± 0.4	
12	**H**	>100	>100	>70		70 ± 5	>10		10 ± 1.2	
RDS1759^c^		2.10	>50							
EFV^c^		0.050 ± 0.012	>0.31							

a Compound concentration required to decrease viral replication in MT4 cells by 50% obtained by three independent experiments (reported as average ± standard deviation).

b Compound concentration required to reduce infected MT4 cells viability by 50% obtained by three independent experiments (reported as average ± standard deviation).

c Data from Corona et al. (2014a).

d Data from Corona et al. (2020).

**Figure 3:**
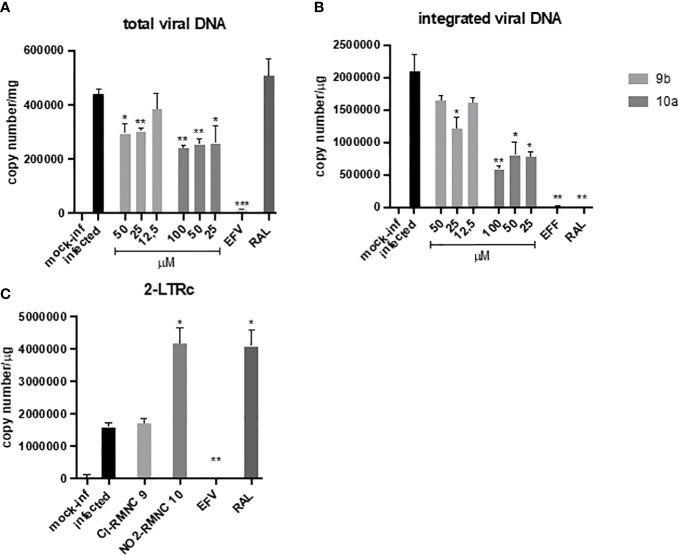
qPCR kinetics of 2-LTRc and integrated DNA forms during a single round of HIV replication in the presence of inhibitors. MT4 cells were infected with HIV-1 in the absence (DMSO) or in the presence of 100 nM of EFV, 500 nM of RAL, and different concentrations of compounds 10a (gray) and 9b (white) added at the time of infection. Samples were analyzed for **(A)** total viral DNA and **(B)** integrated viral DNA at 48 h and **(C)** 2-LTRc by RT-qPCR. Data are represented as mean and standard deviation of two independent experiments. p-Values were calculated between the infected and infected-treated conditions by unpaired, two-tailed t-tests. *p < 0.05; **p < 0.01; ***p < 0.001. DMSO, dimethyl sulfoxide; EFV, efavirenz; RAL, raltegravir.

Accordingly, we measured quantitatively the formation of various viral DNA species during a single HIV-1 replication cycle in the presence of increasing concentrations of 9b and 10a (**Figure 3**), using the NNRTI EFV as a control for retrotranscription and INSTI RAL as a control for integration. Both compounds were able to cause a decrease in total viral DNA, a product of retrotranscription, confirming an inhibitory effect exerted on RT (**Figure 3A**). Moreover, the integrated viral DNA was quantified (**Figure 3B**), on which 10a exerted a significantly more marked effect than 9b.

To verify if this effect was the consequence of the inhibitory activity of 10a against IN, we measured the levels of DNA 2LTRc, a sign of increased events of DNA circularization due to inhibition of its integration into the host genome. A marked increase of DNA 2LTRc was observed in cells treated with 10a, while no increase was observed when 9b was used. Prompted by these interesting results, we investigated the nature of the interaction between 10a and IN in a biochemical assay using RAL as an active site inhibitor and Kuwanon-L operator as positive control (**Table 6**).

**Table 6:** Effect of RMNC compounds on HIV-1 IN activities and dimerization.

NOME	^a^IC_50_ IN LEDGF-dependent integration (μM)	^a^IC_50_ IN LEDGF-independent integration (μM)	^b^IC_50_ IN–IN subunit exchange (μM)	^c^IC_50_ IN/LEDGF binding (μM)
**9b**	>100	>100	>100	>100
**RMNC6 (****Corona et al., 2016b****)**	>100	>100	>100	>100
**1a**	22.35 ± 4.65	100	22.8 ± 2.6	>100
**3a**	15.5 ± 3.0	73.5 ± 13.5	18.5 ± 3.5	>100
**10a**	32.3 ± 2.7	35.5 ± 1.5	11.5 ± 1.5	>100
**RAL**	0.058 ± 0.002	0.061 ± 0.01	>10	>10
**Kuwanon-L**	42 ± 3	34 ± 0.5	^d^38 ± 1.5	22 ± 0.5

a Compound concentration required to reduce HIV-1 IN LEDGF-dependent and LEDGF-independent strand-transfer activity by 50%.

b Compound concentration required to reduce HIV-1 IN subunit exchange by 50%.

c Compound concentration required to reduce HIV-1 IN-LEDGF binding by 50%.

d Compound concentration required to reduce HIV-1 IN multimerization increase by 50% (Esposito, Tintori et al., 2015).

For comparison, 9b was also tested. The results show that, as expected, 9b is not able to inhibit IN. Conversely, 10a acts with a singular mechanism, different from the compounds hitherto known. Indeed, 10a inhibits the enzymatic activity and the interchange of the subunits of IN without interfering with the binding of LEDGF, contrary to Kuwanon-L, which has been predicted to bind the same site but, being a bulkier compound, interferes also with LEDGEF binding site. According to our findings, compound 10a might act by an innovative allosteric mechanism (Shkriabai et al., 2004; Sippel and Sotriffer, 2010), interfering with the IN–IN dimer subunit exchange, which is essential for the enzymatic activity, and the IN multimerization, referred to in literature for other non-catalytic site integrase inhibitors (NCINI), e.g., 1-pyrrolidineacetamide derivatives (Du et al., 2008) (2*E*)-3-[3,4-bis(acetoxy)phenyl]-2-propenoate-*N*-[(2*E*)-3-[3,4-bis(acetyloxy)phenyl]-1-oxo-2-propenyl]-l-serine methyl ester (Kessl et al., 2009). This mechanism is promising because it would offer an option against RAL-resistant strains since most mutations are located close to the DDE motif (Cooper et al., 2008).

Therefore, to better understand the binding of 10a, docking experiments using the enzymes RT and IN were performed.

The interactions between compound 10a (**Figures 2A, B**) and both NNRTI and RNAse H allosteric sites (Himmel et al., 2009) can explain the dual inhibition of both associated catalytic functions. These include π–π with Tyr188, Phe227, Trp229, and Tyr318; a hydrogen bond (HB) with Lys101; and several hydrophobic interactions with Val106, Val108, Phe227, and Trp229.

The complex IN dimer 10a (**Figures 2C, D**) is stabilized by multiple van der Waals contacts with Val88, Ile89, and Pro90 of both dimer chains, as well as cation–π interactions with Lys173 of chains A and B, HB with Tyr99. The acquired knowledge can help to optimize the multitarget activity of this compound and its pharmacokinetic properties.

**Figure 4:**
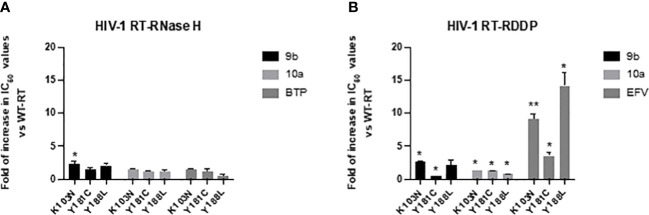
Activity of compounds 9b and 10a against RTs bearing mutations conferring resistance to NNRTIs. Data are represented as mean and standard deviation of two independent experiments. p-Values were calculated between fold increase in IC_50_ compared to wt RT, with two-tailed unpaired t-tests. *p < 0.05; **p < 0.01. RT, reverse transcriptase; NNRTI, non-nucleoside reverse transcriptase inhibitor.

In order to better characterize the potential against circulating drug-resistant variants carrying mutations within the NNRTI binding pocket and close to the binding site predicted for compounds 10a and 9b, both compounds were tested against the RNase H and RDDP activities of HIV-1 RTs bearing K103N, Y181C, and Y188L amino acid substitutions, the most frequent resistance mutations described for NNRTIs (Wensing et al., 2019). For RNase H and RDDP inhibition, compound b-thujaplicinol (BTP) and the NNRTI EFV were used as positive controls, respectively. The results showed that all the enzymes retained almost full sensitivity to both compounds vs. the RT-associated RNase H function, with a slight increase in IC_50_ values that were in all the cases less than threefold and significant only for compound 9b against K103N (**Figure 4A**). A similar result was obtained against the RDDP activity (**Figure 4B**). Also, in this case, the increase in IC_50_, although found to be significant in most of the cases, ranged from 1- to 2.6-fold. For EFV, the increase in IC_50_ values was remarkable, which was 3.5-fold against Y181C, 9.1-fold against K103N, and 14-fold against Y188L. These data suggest that compounds 9b and 10a would retain a good potency of inhibition against circulating variants bearing these mutations.

## Conclusions

These data indicate that the 5-nitro-3-(2-(4-arylthiazol-2-yl)hydrazineylidene)indolin-2-one and 5-chloro-3-(2-(4-arylthiazol-2-yl)hydrazineylidene)indolin-2-one scaffolds have unique potential for the design of both dual RT inhibitors, able to block the replication of HIV-1 acting in an allosteric manner on both catalytic activities of RT, and, for multitarget inhibitors, capable of blocking the replication of HIV-1 by acting in an allosteric manner on both RT and IN. The synthesized molecules have been characterized in detail. The best derivatives undoubtedly deserve further studies to fully characterize the modalities of interaction with the two viral enzymes and the mechanism of action. Structural optimization will hopefully lead to increased inhibitory activity, making a significant step toward the development of a new class of HIV-1 inhibitors.

## Data availability statement

The raw data supporting the conclusions of this article will be made available by the authors without undue reservation.

## Author contributions

All authors listed have made a substantial, direct, and intellectual contribution to the work and approved it for publication.
